# Melioidosis Seroprevalence in Animals: Systematic Review and Meta-Analysis

**DOI:** 10.3390/life16071080

**Published:** 2026-06-27

**Authors:** Jongkonnee Thanasai, Anchalee Chittamma, Supphachoke Khemla, Atthaphong Phongphithakchai, Moragot Chatatikun, Jitbanjong Tangpong, Sa-ngob Laklaeng, Jirarat Songsri, Wiyada Kwanhian Klangbud

**Affiliations:** 1 Faculty of Medicine, Mahasarakham University, Mahasarakham 44000, Thailand; jongkonnee@msu.ac.th; 2 Department of Pathology, Faculty of Medicine Ramathibodi Hospital, Mahidol University, Bangkok 10400, Thailand; anchalee.chi@mahidol.ac.th; 3 Division of Infectious Diseases, Department of Internal Medicine, Nakhon Phanom Hospital, Nakhon Phanom 48000, Thailand; sup.mednkp@gmail.com; 4 Nephrology Unit, Division of Internal Medicine, Faculty of Medicine, Prince of Songkla University, Songkhla 90110, Thailand; atthaphong.p@psu.ac.th; 5 School of Allied Health Sciences, Walailak University, Nakhon Si Thammarat 80160, Thailand; moragot.ch@wu.ac.th (M.C.); rjitbanj@wu.ac.th (J.T.); sumoun2528@gmail.com (S.-n.L.); jirarat.so@wu.ac.th (J.S.); 6 Research Excellence Center for Innovation and Health Products (RECIHP), Walailak University, Nakhon Si Thammarat 80160, Thailand; 7 Medical Technology Program, Faculty of Science, Nakhon Phanom University, Nakhon Phanom 48000, Thailand; 8 Faculty of Medicine, Nakhon Phanom University, Nakhon Phanom 48000, Thailand

**Keywords:** *Burkholderia pseudomallei*, melioidosis, seroprevalence, animals, meta-analysis, One Health, zoonosis

## Abstract

**Background: ***Burkholderia pseudomallei*, the causative agent of melioidosis, infects diverse animal species and reflects environmental contamination. However, the global seroprevalence of *B. pseudomallei* in animals remains incompletely characterized. **Methods:** A systematic review and meta-analysis were conducted according to PRISMA guidelines and registered in PROSPERO (CRD420261306404). PubMed, Embase, and Scopus were searched for observational studies reporting seroprevalence of *B. pseudomallei* in animals. Random-effects meta-analysis was performed to estimate the pooled prevalence with 95% confidence intervals (CIs). Subgroup analyses were conducted by animal group, geographic region, diagnostic method, and indirect hemagglutination assay (IHA) cut-off value. Risk of bias was assessed using the Joanna Briggs Institute checklist. **Results:** Twenty studies involving 78,914 animals were included. The pooled seroprevalence of *B. pseudomallei* was 11% (95% CI: 6–19%), with substantial heterogeneity (*I*^2^ = 98.1%, *p* < 0.0001). Wildlife showed the highest prevalence (16%; 95% CI: 10–25%), followed by livestock (11%; 95% CI: 6–19%). Significant geographic variation was observed (*p* < 0.0001), with higher prevalence reported in North America (18%) and Southeast Asia (10%). Seroprevalence estimates varied according to diagnostic method and IHA cut-off values. Sensitivity analyses yielded similar pooled prevalence estimates after exclusion of small studies, supporting the stability of the overall findings despite persistent heterogeneity. **Conclusions:** Exposure to *B. pseudomallei* is widespread among animal populations and influenced by geographic and methodological factors. Standardized diagnostic approaches and expanded animal surveillance are needed to improve understanding of melioidosis epidemiology within a One Health framework.

## 1. Introduction

Melioidosis is a serious infectious disease caused by *Burkholderia pseudomallei*, an environmental Gram-negative bacterium commonly found in soil and water in tropical and subtropical regions [1,2]. Traditionally, the disease has been associated with endemic areas such as Southeast Asia and northern Australia, but increasing reports from Africa, South Asia, the Caribbean, and the Americas suggest that its geographic range is much wider than previously believed [3,4]. Although melioidosis is mainly recognized as a human health concern because of its high mortality rate, the pathogen also affects many animal species, including livestock, wildlife, and companion animals, reflecting its broad environmental distribution and host adaptability [5,6].

In recent years, animal melioidosis has gained greater attention within the One Health framework, as infected animals may indicate environmental contamination and potential risks for human exposure [6]. However, animal infections are often overlooked because clinical signs are frequently mild, nonspecific, or absent, while accurate diagnosis depends on specialized laboratory testing [2,7]. Although bacterial culture remains the reference standard, serological methods such as IHA, ELISA, and CFT are widely used in epidemiological studies to assess exposure in animal populations [8]. Although previous studies, including Musa et al. (2024), have evaluated global animal melioidosis prevalence [9], important gaps remain regarding the comparative interpretation of seroprevalence estimates across different animal populations, geographic regions, diagnostic assays, and serological cut-off values. Therefore, the present systematic review and meta-analysis aimed to provide an updated quantitative synthesis of animal seroprevalence data and to explore major sources of heterogeneity influencing reported prevalence estimates.

Environmental conditions also strongly influence the transmission of *B. pseudomallei*. Rainfall, flooding, soil characteristics, and farming activities have all been linked to increased disease occurrence in both humans and animals [8,10]. Recent environmental surveillance studies further suggest that climate change and extreme weather events may expand the ecological suitability of the bacterium and increase the risk of infection in new regions [4,11]. Reports of animal melioidosis from previously non-endemic areas further emphasize the need for broader surveillance and a clearer understanding of the pathogen’s global distribution.

Despite growing research interest, important gaps remain. Limmathurotsakul et al. (2016) estimated the global burden and environmental suitability of melioidosis, but the study focused mainly on human disease [1]. Mariappan et al. (2022) highlighted the importance of One Health surveillance, yet their work was largely conceptual and region-specific [6]. Similarly, recent veterinary studies summarized diagnostic and epidemiological findings in animals, but inconsistencies in methods, animal species, and geographic coverage limited direct comparison between studies.

Therefore, this systematic review and meta-analysis aimed to estimate the pooled global seroprevalence of *B. pseudomallei* in animal populations using available serological evidence. The study also explored potential sources of heterogeneity, including animal type, geographic region, diagnostic approach, and serological cut-off values. By integrating evidence from different regions and animal groups within a One Health perspective, this study provides a clearer understanding of animal melioidosis epidemiology and supports future surveillance, diagnostic standardization, and disease control efforts.

## 2. Materials and Methods

### 2.1. Study Design and Registration

This systematic review and meta-analysis was conducted according to a predefined protocol registered in the International Prospective Register of Systematic Reviews (PROSPERO; CRD420261306404) [12]. The review followed the Preferred Reporting Items for Systematic Reviews and Meta-Analyses (PRISMA) guidelines. The objective was to estimate the global seroprevalence of *Burkholderia pseudomallei* in animal populations and to explore potential sources of heterogeneity across studies.

### 2.2. Search Strategy and Eligibility Criteria

A comprehensive literature search was performed across multiple electronic databases using combinations of predefined keywords related to “melioidosis,” “*Burkholderia pseudomallei*,” “seroprevalence,” and “animals.” The search strategies are in Supplementary File S1. Studies were eligible if they reported seroprevalence data in any animal species, used recognized serological diagnostic methods, and provided sufficient quantitative data, including the number of positive cases and the total number of animals tested. Observational study designs, including cross-sectional, cohort, and case–control studies, were considered eligible for inclusion. Studies were excluded if they focused exclusively on human populations, did not report serological outcomes, lacked sufficient quantitative data, or did not clearly describe diagnostic methods or sample characteristics.

### 2.3. Data Extraction and Quality Assessment

Study selection, data extraction, and eligibility assessment were conducted independently by two reviewers. Extracted data included study characteristics such as author, year of publication, geographic region, animal group, diagnostic method, sample size, and number of seropositive cases. Any discrepancies between reviewers were resolved through discussion and consensus. The methodological rigor of included studies was evaluated as part of the overall data synthesis process.

### 2.4. Risk of Bias Assessment

The methodological quality of the included studies was assessed independently by two reviewers using the Joanna Briggs Institute (JBI) Critical Appraisal Checklist for Studies Reporting Prevalence Data [13]. This tool evaluates the risk of bias across nine domains, including the appropriateness of the sample frame, sampling method, adequacy of sample size, description of study subjects and setting, coverage of the identified sample, validity of the diagnostic methods, consistency of measurement across participants, appropriateness of statistical analysis, and adequacy of response rate.

Each item was assessed as “yes,” “no,” “unclear,” or “not applicable.” Studies meeting most JBI domains, particularly those related to sampling methodology, diagnostic validity, and statistical analysis, were classified as low risk of bias. Studies with unclear sampling representativeness or incomplete reporting across several domains were categorized as moderate risk of bias. No studies fulfilled the criteria for high risk of bias across multiple domains. Particular attention was given to sampling methodology and the validity of serological diagnostic assays, as these were identified as critical sources of bias in prevalence studies of *Burkholderia pseudomallei*.

Disagreements between reviewers were resolved through discussion and consensus. The overall risk-of-bias assessment was incorporated into the interpretation of pooled estimates; however, no studies were excluded solely based on risk of bias.

### 2.5. Statistical Analysis

The primary outcome was the pooled seroprevalence of *Burkholderia pseudomallei* in animals, expressed as proportions with corresponding 95% confidence intervals (CI). A random-effects model was applied to account for anticipated between-study heterogeneity. Statistical heterogeneity was evaluated using Cochran’s Q test and the *I*^2^ statistic, with *I*^2^ values greater than 75% considered indicative of substantial heterogeneity.

Subgroup analyses were performed to explore potential sources of heterogeneity according to animal group, geographic region, diagnostic method, and indirect hemagglutination assay (IHA) cut-off values. Sensitivity analyses were conducted by sequentially excluding studies with small sample sizes (<50 animals) to assess the robustness and stability of the pooled prevalence estimates.

Publication bias and small-study effects were assessed through visual inspection of funnel plot asymmetry and quantitatively using Egger’s linear regression test [14]. The regression analysis was performed using inverse-variance weighting with standard error as the predictor variable. A *p*-value < 0.05 was considered statistically significant. Meta-analyses were performed in R (version 4.4.2) using the meta package. Pooled prevalence estimates were calculated using the *metaprop* function with logit-transformed proportions (PLOGIT). Between-study variance was estimated using the DerSimonian–Laird method. Studies reporting zero seropositive animals were retained in the analysis using the continuity correction implemented within the *metaprop* procedure. A random-effects model was applied throughout because substantial between-study heterogeneity was anticipated.

## 3. Results

### 3.1. Study Selection

The literature search identified a total of 670 records from electronic databases, including PubMed (*n* = 222), Embase (*n* = 328), and Scopus (*n* = 120). After removal of 365 duplicate records, 305 studies remained for title and abstract screening. During the screening process, 277 records were excluded, including review articles and systematic reviews (*n* = 34), in vitro studies (*n* = 5), and studies unrelated to serodiagnosis (*n* = 238).

**Figure 1:**
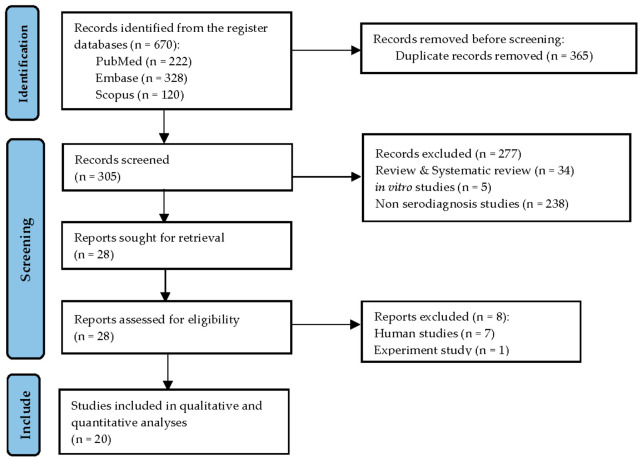
PRISMA flow diagram.

A total of 28 full-text articles were assessed for eligibility. Of these, eight full-text articles were excluded, including seven studies involving human populations and one experimental animal study. Ultimately, 20 studies met the inclusion criteria and were included in both the qualitative and quantitative syntheses. The study selection process is summarized in the PRISMA flow diagram (Figure 1).

### 3.2. Characteristics of Included Studies

A total of 20 studies published between 1972 and 2025 were included in the meta-analysis, comprising 78,914 animals from multiple geographic regions, including Southeast Asia, North America, Africa, and Oceania. The included studies investigated a variety of animal populations, including livestock, wildlife, companion animals, and military animals. Military animals were categorized separately because of their unique environmental exposure and occupational deployment conditions. Livestock represented the majority of the studied populations and included cattle, goats, pigs, sheep, and horses, whereas wildlife studies involved species such as macaques and other free-ranging animals.

Various serological methods were used to detect antibodies against *Burkholderia pseudomallei*, including indirect hemagglutination assay (IHA), enzyme-linked immunosorbent assay (ELISA), complement fixation test (CFT), GLANDA-ELISA, and combined CFT/IHA approaches. Considerable variation was observed in diagnostic cut-off values, particularly for IHA, with thresholds ranging from 1:40 to 1:320. Sample sizes varied substantially across studies, ranging from fewer than 20 animals to more than 70,000 animals. Detailed characteristics of the included studies are summarized in Table 1.

### 3.3. Overall Seroprevalence

**Figure 2:**
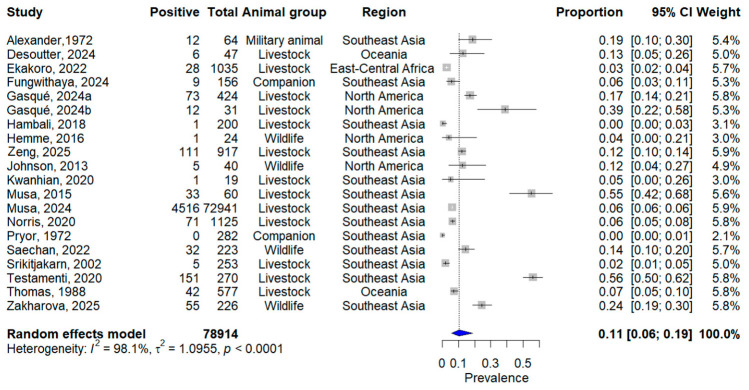
Forest plot of the pooled prevalence of 20 studies [8,15,16,17,18,19,20,21,22,23,24,25,26,27,28,29,30,31,32,33]. Squares represent the prevalence estimates from individual studies, with square size proportional to study weight. Horizontal lines indicate the corresponding 95% confidence intervals (CIs). The blue diamond represents the pooled prevalence estimate and its 95% CI derived from the random-effects model.

The pooled global seroprevalence of *Burkholderia pseudomallei* in animal populations was estimated at 11% (95% CI: 6–19%) under a random-effects model (Figure 2). There was substantial heterogeneity across studies, as indicated by an *I*^2^ value of 98.1% (*p* < 0.0001), suggesting considerable variability in prevalence estimates across different study settings and populations.

### 3.4. Subgroup Analysis by Animal Group

**Figure 3:**
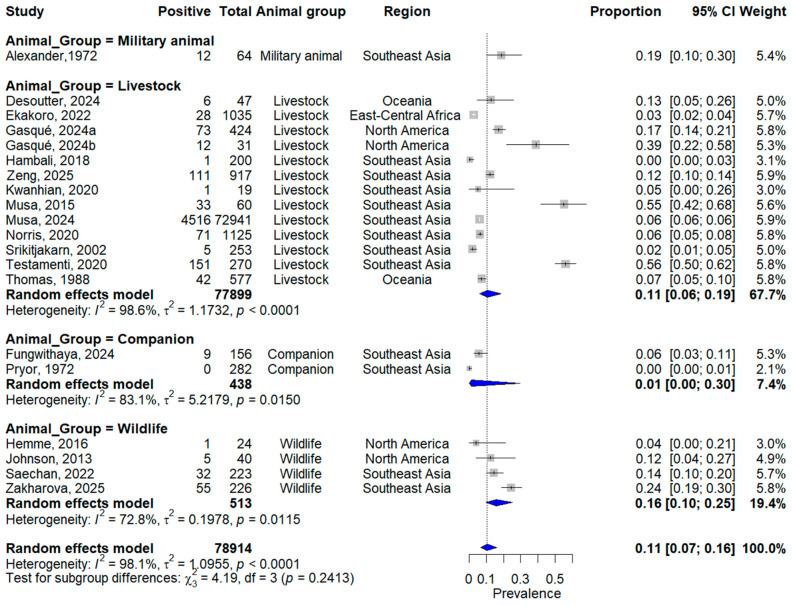
Forest plot of seroprevalence according to animal group [8,15,16,17,18,19,20,21,22,23,24,25,26,27,28,29,30,31,32,33]. Squares represent the prevalence estimates from individual studies, with square size proportional to study weight. Horizontal lines indicate the corresponding 95% confidence intervals (CIs). The blue diamond represents the pooled prevalence estimate and its 95% CI derived from the random-effects model.

Subgroup analysis based on animal group showed variation in seroprevalence estimates, as shown in Figure 3. Livestock demonstrated a pooled prevalence of 11% (95% CI: 6–19%), while wildlife exhibited a higher prevalence of 16% (95% CI: 10–25%). Companion animals showed a markedly lower pooled prevalence of 1% (95% CI: 0–30%), although this estimate was based on limited data and wide confidence intervals. A single study involving military animals reported a prevalence of 19%. Despite these variations, statistical analysis indicated no significant differences between animal groups (*p* = 0.2413), suggesting that host type alone did not fully explain the observed heterogeneity.

### 3.5. Subgroup Analysis by Geographic Region

**Figure 4:**
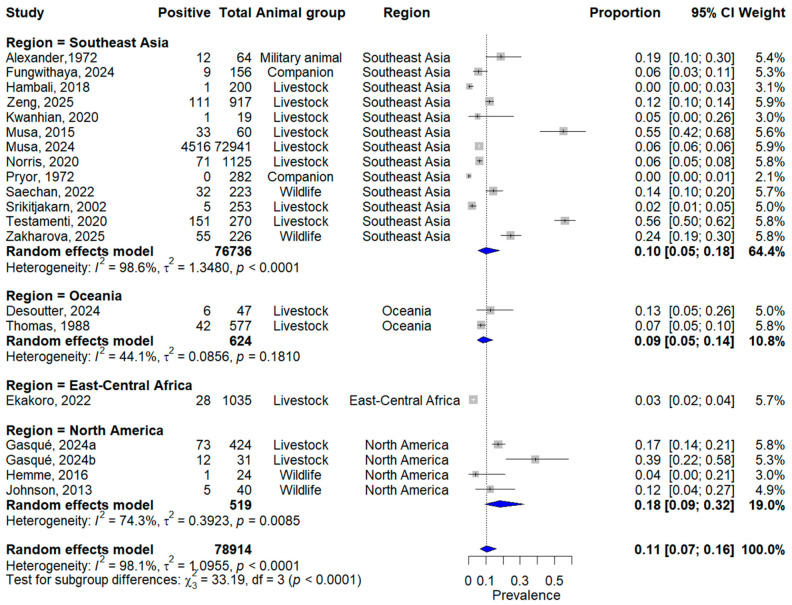
Forest plot of the subgroup by geographic region [8,15,16,17,18,19,20,21,22,23,24,25,26,27,28,29,30,31,32,33]. Squares represent the prevalence estimates from individual studies, with square size proportional to study weight. Horizontal lines indicate the corresponding 95% confidence intervals (CIs). The blue diamond represents the pooled prevalence estimate and its 95% CI derived from the random-effects model.

Geographic variation in seroprevalence was evident across studies, as shown in Figure 4. North America showed the highest pooled prevalence (18%; 95% CI: 9–32%), followed by Southeast Asia (10%; 95% CI: 5–18%), Oceania (9%; 95% CI: 5–14%), and Africa (3%; 95% CI: 2–4%). Subgroup differences across regions were statistically significant (*p* < 0.0001), indicating that geographic factors play a major role in influencing seroprevalence.

### 3.6. Subgroup Analysis by Diagnostic Method

**Figure 5:**
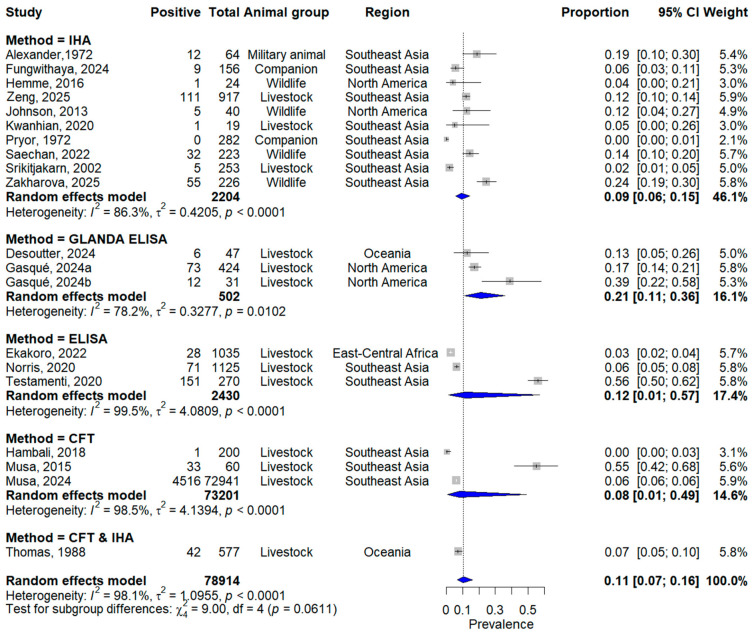
Forest plot of the subgroup by diagnostic method [8,15,16,17,18,19,20,21,22,23,24,25,26,27,28,29,30,31,32,33]. Squares represent the prevalence estimates from individual studies, with square size proportional to study weight. Horizontal lines indicate the corresponding 95% confidence intervals (CIs). The blue diamond represents the pooled prevalence estimate and its 95% CI derived from the random-effects model.

Seroprevalence estimates varied according to the diagnostic method used, as shown in Figure 5. Studies employing IHA reported a pooled prevalence of 9% (95% CI: 6–15%), while ELISA-based studies showed a prevalence of 12% (95% CI: 1–57%). Studies using CFT reported a prevalence of 8% (95% CI: 1–49%), and GLANDA-ELISA yielded a higher estimate of 21% (95% CI: 11–36%). However, subgroup differences according to diagnostic method did not reach statistical significance (*p* = 0.0611). Although prevalence estimates varied numerically across assays, these findings should be interpreted cautiously, as the observed differences may reflect methodological variability and limited study numbers within some diagnostic categories rather than true differences in seroprevalence.

### 3.7. Subgroup Analysis by IHA Cut-Off Values

**Figure 6:**
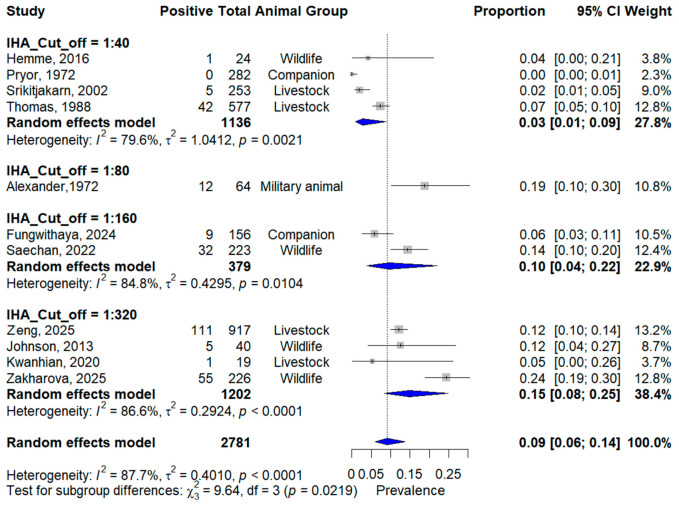
Forest plot of the subgroup by IHA cut-off [15,18,22,23,24,25,28,29,30,31,32,33]. Squares represent the prevalence estimates from individual studies, with square size proportional to study weight. Horizontal lines indicate the corresponding 95% confidence intervals (CIs). The blue diamond represents the pooled prevalence estimate and its 95% CI derived from the random-effects model.

Analysis based on IHA cut-off values demonstrated significant variability in seroprevalence estimates, as shown in Figure 6. Studies using a cut-off of 1:80 reported a prevalence of 19%, whereas those using a cut-off of 1:160 reported a prevalence of 10%. Lower cut-off values, such as 1:40, yielded lower prevalence estimates of 3%, whereas higher cut-offs, such as 1:320, were associated with a prevalence of 15%. The differences between these subgroups were statistically significant (*p* = 0.0219), indicating that diagnostic thresholds have a substantial impact on reported seroprevalence.

### 3.8. Sensitivity Analysis

**Figure 7:**
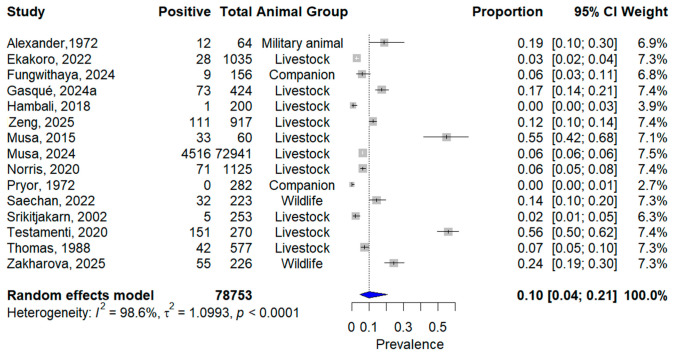
Forest plot of 15 studies [8,15,17,18,19,21,23,26,27,28,29,30,31,32,33] after exclusion of studies with sample sizes < 50 animals [16,20,22,24,25]. Squares represent the prevalence estimates from individual studies, with square size proportional to study weight. Horizontal lines indicate the corresponding 95% confidence intervals (CIs). The blue diamond represents the pooled prevalence estimate and its 95% CI derived from the random-effects model.

Sensitivity analyses were conducted to assess the robustness of the findings. Exclusion of studies with sample sizes < 50 animals did not materially alter the pooled estimate (*I*^2^ = 98.6%, *p* < 0.0001), indicating that the findings were robust to the removal of small studies. Substantial heterogeneity, however, remained. The corresponding forest plot is shown in Figure 7.

### 3.9. Publication Bias

Assessment of publication bias using Egger’s regression test suggested possible small-study effects (t = 1.99, *p* = 0.0619), although the result did not reach statistical significance. Potential small-study effects with possible funnel plot asymmetry were observed. However, this effect diminished after sensitivity analyses excluding smaller studies (<50), *p*-values of 0.0990 (Supplementary File S2). These findings suggest that the observed funnel plot asymmetry may be more consistent with small-study effects than with substantial publication bias. Smaller studies are more likely to produce extreme prevalence estimates because of reduced precision and increased sampling variability.

### 3.10. Quality of Included Studies

Based on the JBI checklist, the overall methodological quality of included studies ranged from low to moderate risk of bias. Most studies were classified as having a low risk of bias due to adequate sample size, clear description of study populations, and appropriate statistical analyses. However, several studies were classified as moderate risk, primarily due to limitations in sampling methodology and representativeness.

The most common source of bias was the use of non-probability sampling methods, which may limit generalizability. Smaller studies were also associated with increased uncertainty and reduced precision. In contrast, most studies used validated serological methods, although variability in assay types and cut-off values introduces potential measurement bias.

Reporting of response rates was frequently unclear across studies, preventing full assessment of non-response bias. Despite these limitations, the overall quality of evidence was considered sufficient to support meta-analysis, although findings should be interpreted in light of the identified methodological constraints. Detailed quality scores for each study are provided in Supplementary Table S1.

## 4. Discussion

This systematic review and meta-analysis synthesized evidence from 20 studies involving 78,914 animals to estimate the global seroprevalence of *Burkholderia pseudomallei* in animals. The pooled seroprevalence of 11% (95% CI: 6–19%) suggests that exposure to the pathogen is relatively common among animal populations and further supports the role of animals as indicators of environmental contamination. However, this estimate should be interpreted cautiously because substantial between-study heterogeneity (*I*^2^ = 98.1%) indicates considerable variability across animal populations, geographic settings, diagnostic methods, serological cut-off values, sampling strategies, and study methodologies. Therefore, the pooled estimate should primarily be considered a broad summary measure rather than a precise estimate of global epidemiological burden. In addition, funnel plot asymmetry and persistent residual heterogeneity observed after sensitivity analyses suggest that small-study effects and methodological variability may also have influenced the pooled estimates. These findings are consistent with the ecological nature of *B. pseudomallei* as an environmental bacterium capable of persisting in soil and water and infecting a broad range of hosts across different ecological settings [2,5]. The high level of heterogeneity observed among studies indicates that seroprevalence patterns are likely influenced by multiple interacting factors, including geography, environmental conditions, animal species, and diagnostic approaches, rather than random variation alone. Similar observations have been reported in previous One Health investigations, which emphasized the complexity of melioidosis epidemiology at the human–animal–environment interface [6]. Differences in study design and sampling strategies likely contributed substantially to variability between studies. Included investigations ranged from small cross-sectional surveys [16,20,22,24,25] to large surveillance studies [8,15,18,19,21,23,27,30,31,32,33] involving highly diverse animal populations and ecological settings. Variations in animal husbandry, wildlife exposure, environmental conditions, and targeted surveillance approaches may influence the probability of detecting seropositive animals. In addition, differences in serological assays, antigen targets, and diagnostic cut-off values reduce direct comparability between studies and may partly explain the substantial heterogeneity observed across pooled estimates.

The findings of this review are broadly consistent with previous literature, suggesting that melioidosis is more geographically widespread than historically recognized. While Southeast Asia and northern Australia remain the best-known endemic regions, increasing reports from Africa and North America have challenged the traditional understanding of the disease distribution [3,4]. Although higher pooled prevalence estimates were observed in North America, these findings should be interpreted cautiously because they were derived from a limited number of studies and may not fully represent the broader regional epidemiology. Recent reports from travelers and imported animal cases have identified melioidosis in regions previously considered non-endemic [34]. These observations may partly reflect improved surveillance and diagnostic capacity, but they may also indicate genuine geographic expansion associated with environmental and climatic changes.

The subgroup analyses also suggested higher seroprevalence among wildlife species compared with livestock and companion animals, although subgroup differences did not reach statistical significance. Wildlife likely experiences greater environmental exposure because of unrestricted contact with natural soil and water sources that may harbor *B. pseudomallei*. Previous ecological and environmental studies have similarly demonstrated widespread bacterial persistence in wet soils, tropical environments, and flood-prone areas [10]. The detection of substantial seropositivity among apparently healthy wildlife species may indicate that subclinical exposure is common in endemic environments. In contrast, lower prevalence estimates among companion animals may reflect reduced environmental contact under controlled living conditions. However, interpretation should remain cautious because differences in sample size, species diversity, and study methodology may also contribute to variation between animal groups.

Environmental and management factors appeared to play important roles in shaping exposure risk. Several included studies reported associations between seropositivity and rainfall, flooding, soil moisture, grazing systems, and farming practices. These findings align with previous epidemiological evidence showing that climatic and ecological conditions strongly influence the environmental survival and transmission dynamics of *B. pseudomallei* [8]. Heavy rainfall and flooding may facilitate the dissemination of the organism through contaminated soil and surface water, thereby increasing opportunities for animal exposure. Similarly, free-grazing livestock may encounter contaminated environmental sources more frequently than intensively managed animals. Increasing evidence also suggests that climate change and extreme weather events may expand the ecological suitability of *B. pseudomallei*, potentially contributing to the emergence of melioidosis in new geographic areas [4,11].

Interestingly, studies using GLANDA-ELISA demonstrated numerically higher pooled seroprevalence estimates compared with other diagnostic methods. However, this assay was originally developed for serological detection of *Burkholderia mallei* [35] and may therefore demonstrate different antigenic characteristics or potential cross-reactivity when applied to *Burkholderia pseudomallei* surveillance. Consequently, the higher prevalence observed in GLANDA-ELISA studies may reflect differences in assay sensitivity, specificity, antigenic targets, or regional epidemiological characteristics of the included study populations. Nevertheless, interpretation should remain cautious because only a limited number of GLANDA-ELISA studies were available. Although subgroup differences by diagnostic method were not statistically significant, prevalence estimates varied considerably between assays. Previous studies have similarly highlighted the lack of standardized serological methods for melioidosis surveillance and the potential impact of assay selection on prevalence estimates [22,30]. In addition, significant differences according to IHA cut-off values suggest that diagnostic thresholds substantially influence reported seroprevalence. Lower cut-off values may increase sensitivity but also increase the likelihood of cross-reactivity and false-positive results, whereas higher thresholds may improve specificity while underestimating prior exposure. Previous studies have reported cross-reactivity between melioidosis and glanders serological assays, highlighting the challenges associated with serological interpretation.

Another important consideration is that seropositivity reflects previous exposure rather than confirmed active infection. Antibody responses to *B. pseudomallei* may persist for prolonged periods and may occur in the absence of clinical disease, particularly in endemic settings where repeated environmental exposure is possible [1]. Consequently, serological evidence should not be interpreted as a direct indicator of disease burden. This distinction is particularly relevant in wildlife populations, where asymptomatic exposure may be relatively common and where surveillance often relies exclusively on serological screening. The complexity of interpreting antibody responses may therefore contribute to variability among studies and complicate direct comparisons across geographic regions and animal populations.

Despite these limitations, the overall findings remained relatively robust. Sensitivity analyses demonstrated that exclusion of smaller studies did not materially alter pooled prevalence estimates, suggesting that the results were not driven solely by small-study effects. Although Egger’s regression test initially suggested possible small-study effects, this association weakened after exclusion of smaller studies. In addition, most included studies were not classified as high risk of bias according to the methodological quality assessment, supporting the overall reliability of the synthesized evidence despite limitations related to sampling representativeness and diagnostic standardization.

Several limitations should nevertheless be acknowledged. The substantial heterogeneity observed across studies limits the precision and generalizability of pooled estimates. Many studies relied on non-probability sampling approaches, which may introduce selection bias and reduce the representativeness of the target populations. Variability in serological assays and diagnostic cut-off values may also have introduced measurement bias and complicated direct comparisons between studies. Furthermore, geographic coverage remained uneven, with limited data from several potentially endemic regions. As a result, the true global burden and distribution of animal melioidosis may still be underestimated. Because most included studies were cross-sectional, causal relationships between environmental exposures and seropositivity could not be established.

Although Egger’s regression test suggested possible funnel plot asymmetry, the result did not reach statistical significance and became less pronounced after exclusion of smaller studies. This finding suggests that small-study effects, rather than true publication bias, may partially explain the observed asymmetry. Smaller prevalence studies are more likely to report extreme estimates because of limited precision and sampling variability, particularly in heterogeneous epidemiological settings. Therefore, while publication bias cannot be completely excluded, the stability of pooled estimates during sensitivity analyses supports the overall robustness and reliability of the findings.

Future research should prioritize the development of standardized diagnostic protocols and harmonized serological cut-off values to improve comparability across epidemiological studies. Longitudinal investigations are needed to better understand antibody persistence, temporal exposure patterns, and the relationship between seropositivity and active infection. Expanded surveillance in underrepresented regions, particularly Africa, would strengthen understanding of the global epidemiology of animal melioidosis. Prevalence estimates from regions represented by limited datasets, especially North America, should be interpreted cautiously because they are based on a small number of studies and may not fully reflect broader regional epidemiology. Integrating environmental monitoring, molecular epidemiology, and animal surveillance within a One Health framework may further clarify transmission pathways and ecological drivers of pathogen persistence.

This study provides an updated global synthesis of *Burkholderia pseudomallei* seroprevalence in animals and highlights important epidemiological and methodological sources of heterogeneity. The findings demonstrate widespread environmental exposure among animal populations and reinforce the value of animal surveillance within One Health monitoring systems. Although the results should be interpreted cautiously because of substantial heterogeneity and diagnostic variability, the evidence underscores the global importance of animal melioidosis and the need for improved surveillance, standardized diagnostic approaches, and interdisciplinary collaboration to advance understanding and control of this neglected infectious disease.

## 5. Conclusions

This systematic review and meta-analysis demonstrated that exposure to *Burkholderia pseudomallei* is widespread among animal populations, with an overall pooled seroprevalence of 11%. Significant variability was observed across geographic regions, animal groups, diagnostic methods, and IHA cut-off values, highlighting the complex epidemiology of animal melioidosis. Wildlife and livestock populations demonstrated numerically higher pooled seroprevalence estimates compared with companion animals, although subgroup differences were not statistically significant.

The substantial heterogeneity identified across studies emphasizes the need for standardized serological methods and harmonized diagnostic criteria to improve comparability between studies. Environmental factors, including rainfall, flooding, and soil exposure, appear to play important roles in transmission dynamics and may contribute to regional differences in prevalence.

Despite methodological limitations and uneven geographic coverage, this study provides an updated global quantitative synthesis of *B. pseudomallei* seroprevalence in animals and further explores important epidemiological and methodological sources of heterogeneity. Expanded surveillance, particularly in underrepresented regions, together with improved diagnostic standardization and longitudinal epidemiological studies, will be essential for strengthening the understanding of animal melioidosis and supporting future disease prevention and control strategies.

## Figures and Tables

**Table 1:** Characteristics of 20 included studies.

Study	[Ref]	Country	Population or Species	*n* Total	*n* Positive	Diagnostic Method (Cutoff)	Qualitative Findings and Risk Factors
Alexander, 1972	[15]	South Vietnam	Military Scout and Tracker Dogs	64	12	IHA (≥1:80)	Prevalence linked to length of service in endemic areas.
Desoutter, 2024	[16]	New Caledonia	Goats	47	6	GLANDA ELISA	Identified ST2045; first confirmed animal case in the region.
Ekakoro, 2022	[17]	Uganda	Market Pigs	1035	28	OPS-B ELISA	Seropositivity is significantly higher during rainy months.
Fungwithaya, 2024	[18]	Thailand	Sheltered Dogs	156	9	IHA (≥1:160)	Coinfection with blood pathogens significantly reduces platelet counts.
Gasqué, 2024a	[19]	French Guiana	Cattle/Horses	361/63	58/15	GLANDA ELISA	Animals used as sentinels for the local establishment of *B. pseudomallei.*
Gasqué, 2024b	[20]	Guadeloupe	Goats	31	12	GLANDA ELISA	Identified ST92 in soil/goats, matching regional human cases.
Hambali, 2018	[21]	Malaysia	Goats/Sheep	100/100	1/0	CFT (≥1:8)	Semi-intensive management and moist clay soil increase risk.
Hemme, 2016	[22]	Puerto Rico	Patas and Rhesus Monkeys	24	1	IHA (≥1:40)	Evidence of exposure in feral monkey populations without travel history.
Zeng, 2025	[23]	Lao PDR	Cattle/Buffalo/Swine	281/137/499	64/27/20	IHA (≥1:320)	Significant spatial cluster identified in Savannakhet province.
Johnson, 2013	[24]	USA (Imported)	Pigtail Macaques	40	5	IHA (≥1:320)	Risk of subclinical/latent infection in macaques imported from Indonesia.
Kwanhian, 2020	[25]	Thailand	Pig Sows (Breeding Farm)	19	1	IHA (≥1:320)	Outbreak linked to contaminated groundwater; identified ST392.
Musa, 2015	[26]	Malaysia	Small Ruminant Farms	60	33	CFT (≥1:80)	Risk factors: Flooding (OR = 11.95) and bush clearing (OR = 6.61).
Musa, 2024	[27]	Malaysia	Total Livestock	72,941	4516	CFT (≥1:80)	Strong positive correlation between seroprevalence and monthly rainfall.
Norris, 2020	[8]	Vietnam	Swine	1125	71	OPS/Hcp1 ELISA	Grazing pigs (11.4%) had higher exposure than farmed pigs (4%).
Pryor, 1972	[28]	Thailand/Vietnam	Sentry Dogs	232/50	0/0	IHA (≥1:40)	No antibodies detected in dogs kept in well-maintained military bases.
Saechan, 2022	[29]	Thailand	Long-tailed Macaques	223	32	IHA (≥1:160)	Monkeys free-living near urban areas can act as reservoirs.
Srikitjakarn, 2002	[30]	Thailand	Dairy Cattle	253	5	IHA (≥1:40)	Prevalence is lower in the North (2%) compared to Northeast Thailand.
Testamenti, 2020	[31]	Indonesia	Macaques	270	151	LPS/Protein ELISA	Facilities with soil flooring had the highest seropositivity.
Thomas, 1988	[32]	Australia	Goats	577	42	CFT (≥1:8)/IHA (≥1:40)	IHA-A is best for screening; CFT is most specific for active infection.
Zakharova, 2025	[33]	Vietnam	Asiatic Black Bears	226	55	IHA (≥1:320)	Non-clonal outbreak involving at least two distinct infection sources.

CFT: complement fixation test, ELISA: Enzyme-linked immunosorbent assay, Hcp1: hemolysin co-regulated protein 1, IHA: indirect hemagglutination assay, Lao PDR: Lao People’s Democratic Republic, LPS: lipopolysaccharide, OPS: O-antigen Polysaccharide, OR: odd ratio, USA: United States of America.

## Data Availability

No new data were created or analyzed in this study. Data sharing is not applicable to this article.

## Supplementary Materials

The following supporting information can be downloaded at: https://www.mdpi.com/article/10.3390/life16071080/s1, Supplementary File S1: Search Strategies; Supplementary File S2: Funnel plot and linear regression; Supplementary Table S1: Quality of studies by Joanna Briggs Institute (JBI) assessment.

## Abbreviations

The following abbreviations are used in this manuscript:

CI	Confidence interval
CFT	Complement fixation test
ELISA	Enzyme-linked immunosorbent assay
Hcp1	hemolysin co-regulated protein 1
IHA	Indirect hemagglutination assay
Lao PDR	Lao People’s Democratic Republic
LPS	Lipopolysaccharide
OPS	O-antigen polysaccharide
OR	Odd ratio
USA	United States of America
