# Supplementary material for: Melioidosis Seroprevalence in Animals: Systematic Review and Meta-Analysis
Source: Life (Basel). 2026 Jun 27;16(7):1080. doi: 10.3390/life16071080 (PMC13413383; doi:10.3390/life16071080)
Supplement: Supplementary file 1 [file life-16-01080-s001.zip › Supplementary File S2_Funnel plot and linear regression.pdf]

Melioidosis Seroprevalence in Animals: Systematic Review and Meta-analysis

Jongkonnee Thanasai [1], Anchalee Chittamma [2], Supphachoke Khemla [3], Atthaphong Phongphithakchai [4], Moragot Chatatikun [5,6], Jitbanjong Tangpong [5,6], Sa-ngob Laklaeng [5], Jirarat Songsri [5] and Wiyada Kwanhian Klangbud [7,8,*]

1 Faculty of Medicine, Mahasarakham University, Mahasarakham 44000, Thailand; jongkonnee@msu.ac.th
2 Department of Pathology, Faculty of Medicine Ramathibodi Hospital, Mahidol University, Bangkok 10400, Thailand; anchalee.chi@mahidol.ac.th
3 Division of Infectious Diseases, Department of Internal Medicine, Nakhon Phanom Hospital,
Nakhon Phanom 48000, Thailand; sup.mednkp@gmail.com
4 Nephrology Unit, Division of Internal Medicine, Faculty of Medicine, Prince of Songkla University, Songkhla 90110, Thailand; atthaphong.p@psu.ac.th
5 School of Allied Health Sciences, Walailak University, Nakhon Si Thammarat 80160, Thailand; moragot.ch@wu.ac.th (M.C.); rjitbanj@wu.ac.th (J.T.); sumoun2528@gmail.com (S.-n.L.); jirarat.so@wu.ac.th (J.S.)
6 Research Excellence Center for Innovation and Health Products (RECIHP), Walailak University,
Nakhon Si Thammarat 80160, Thailand; moragot.ch@wu.ac.th (M.C.); rjitbanj@wu.ac.th (J.T.)
7 Medical Technology Program, Faculty of Science, Nakhon Phanom University,
Nakhon Phanom 48000, Thailand; wiyadakwanhian@gmail.com
8 Faculty of Medicine, Nakhon Phanom University, Nakhon Phanom 48000, Thailand; wiyadakwanhian@gmail.com
* Correspondence: wiyadakwanhian@gmail.com

Supplementary File S2. Funnel plots and Linear regression test

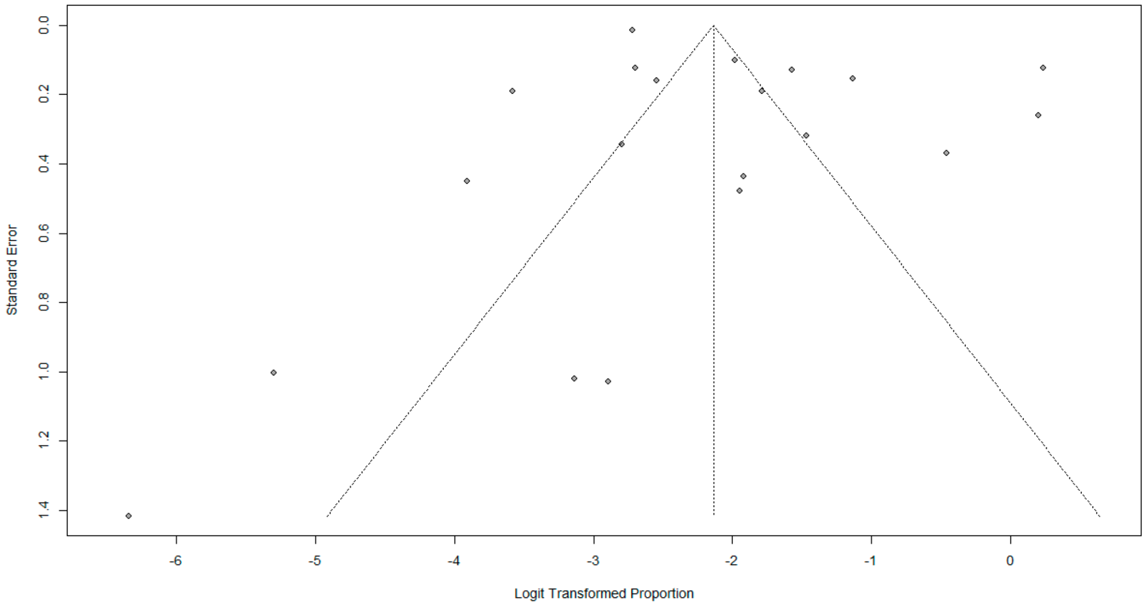

Linear regression test of funnel plot asymmetry

Test result: t = 1.99, df = 18, p-value = 0.0619
Bias estimate: 3.4416 (SE = 1.7285)

Details:
- multiplicative residual heterogeneity variance (tau^2 = 46.4391)
- predictor: standard error
- weight: inverse variance

Sensitivity (Remove n<50)

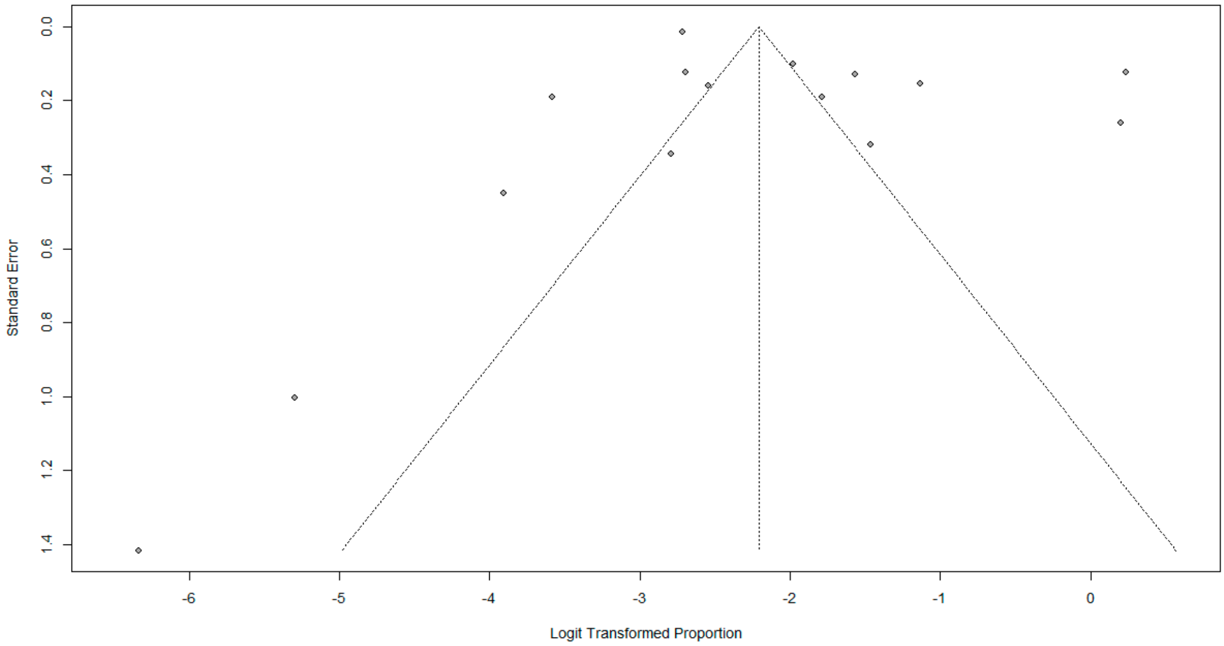

Linear regression test of funnel plot asymmetry

Test result: t = 1.78, df = 13, p-value = 0.0990
Bias estimate: 4.1621 (SE = 2.3422)

Details:
- multiplicative residual heterogeneity variance (tau^2 = 60.7069)
- predictor: standard error
- weight: inverse variance
