# Supplementary material for: Melioidosis Seroprevalence in Animals: Systematic Review and Meta-Analysis
Source: Life (Basel). 2026 Jun 27;16(7):1080. doi: 10.3390/life16071080 (PMC13413383; doi:10.3390/life16071080)
Supplement: Supplementary file 1 [file life-16-01080-s001.zip › Supplementary Table S1_JBI Assessment.pdf]

Melioidosis Seroprevalence in Animals: Systematic Review and Meta-analysis

Jongkonnee Thanasai [1], Anchalee Chittamma [2], Supphachoke Khemla [3], Atthaphong Phongphithakchai [4], Moragot Chatatikun [5,6], Jitbanjong Tangpong [5,6], Sa-ngob Laklaeng [5], Jirarat Songsri [5] and Wiyada Kwanhian Klangbud [7,8,*]

[1] Faculty of Medicine, Mahasarakham University, Mahasarakham 44000, Thailand; jongkonnee@msu.ac.th
[2] Department of Pathology, Faculty of Medicine Ramathibodi Hospital, Mahidol University, Bangkok 10400, Thailand; anchalee.chi@mahidol.ac.th
[3] Division of Infectious Diseases, Department of Internal Medicine, Nakhon Phanom Hospital, Nakhon Phanom 48000, Thailand; sup.mednkp@gmail.com
[4] Nephrology Unit, Division of Internal Medicine, Faculty of Medicine, Prince of Songkla University, Songkhla 90110, Thailand; atthaphong.p@psu.ac.th
[5] School of Allied Health Sciences, Walailak University, Nakhon Si Thammarat 80160, Thailand; moragot.ch@wu.ac.th (M.C.); rjitbanj@wu.ac.th (J.T.); sumoun2528@gmail.com (S.-n.L.); jirarat.so@wu.ac.th (J.S.)
[6] Research Excellence Center for Innovation and Health Products (RECIHP), Walailak University, Nakhon Si Thammarat 80160, Thailand; moragot.ch@wu.ac.th (M.C.); rjitbanj@wu.ac.th (J.T.)
[7] Medical Technology Program, Faculty of Science, Nakhon Phanom University, Nakhon Phanom 48000, Thailand; wiyadakwanhian@gmail.com
[8] Faculty of Medicine, Nakhon Phanom University, Nakhon Phanom 48000, Thailand; wiyadakwanhian@gmail.com
* Correspondence: wiyadakwanhian@gmail.com

Supplementary Table S1. JBI assessment of 20 studies

**Study**	**Frame**	**Sampling**	**Size**	**Description**	**Coverage**	**Valid Method**	**Measurement**	**Analysis**	**Response**	**Overall**
Alexander, 1972	U	N	N	Y	U	Y	Y	Y	U	Moderate
Desoutter, 2024	Y	U	Y	Y	Y	Y	Y	Y	U	Low
Ekakoro, 2022	Y	U	Y	Y	Y	Y	Y	Y	U	Low
Fungwithaya, 2024	Y	U	Y	Y	Y	Y	Y	Y	U	Low

Study	Frame	Sampling	Size	Description	Coverage	Valid Method	Measurement	Analysis	Response	Overall
Gasqué, 2024a	Y	U	Y	Y	Y	Y	Y	Y	U	Low
Gasqué, 2024b	Y	U	N	Y	U	Y	Y	Y	U	Moderate
Hambali, 2018	U	N	Y	Y	U	Y	Y	Y	U	Moderate
Hemme, 2016	U	N	N	Y	U	Y	Y	Y	U	Moderate
Zeng, 2025	Y	U	Y	Y	Y	Y	Y	Y	U	Low
Ismail, 2024	Y	U	Y	Y	Y	Y	Y	Y	U	Low
Johnson, 2013	U	N	N	Y	U	Y	Y	Y	U	Moderate
Kwanhian, 2020	U	N	N	Y	U	Y	Y	Y	U	Moderate
Musa, 2015	U	N	Y	Y	U	Y	Y	Y	U	Moderate
Norris, 2020	Y	U	Y	Y	Y	Y	Y	Y	U	Low
Pryor, 1972	U	N	Y	Y	U	Y	Y	Y	U	Moderate
Saechan, 2022	Y	U	Y	Y	Y	Y	Y	Y	U	Low
Sritjakarn, 2002	Y	U	Y	Y	Y	Y	Y	Y	U	Low
Testamenti, 2020	Y	U	Y	Y	Y	Y	Y	Y	U	Low

Study	Frame	Sampling	Size	Description	Coverage	Valid Method	Measurement	Analysis	Response	Overall
Thomas, 1988	U	N	Y	Y	U	Y	Y	Y	U	Moderate
Zakharova, 2025	Y	U	Y	Y	Y	Y	Y	Y	U	Low

Key for JBI Appraisal Questions:

- Q1: Was the sample representative of the target population?
- Q2: Were study participants recruited in an appropriate way?
- Q3: Was the sample size adequate? (Rated "N" for studies with as flagged in sensitivity tests).
- Q4: Were the study subjects and setting described in detail?
- Q5: Is the data analysis conducted with sufficient coverage of the identified sample?
- Q6: Were valid methods used for identification (e.g., standard serology: IHA, ELISA, CFT)?
- Q7: Was the condition measured in a standard, reliable way for all participants?
- Q8: Was there appropriate statistical analysis?
- Q9: Was the response rate adequate, and if not, was it managed appropriately?

(Ratings: Y = Yes, N = No, U = Unclear)
